# Comparison of Transatlantic Assertive Community Teams (ACT) and Assertive Outreach Teams (AOT) Services

**DOI:** 10.1192/bjo.2026.11606

**Published:** 2026-06-30

**Authors:** Ashish Pathak, Pranveer Singh, Abu Abraham, Laxmikanth Bangaru, Vijay Natarajan

**Affiliations:** 1 Essex Partnership Univeristy NHS Foundation Trust, Basildon, United Kingdom; 2 Essex Partnership Univeristy NHS Foundation Trust, Rochford, United Kingdom; 3 Sussex Partnership NHS Foundation Trust, East Grinstead, United Kingdom; 4 Vancouver Assesrtive Community Treatment Teams, Vancouver, Canada

## Abstract

**Aims:**

To compare UK Assertive Outreach Teams (AOT) and Vancouver Assertive Community Treatment (ACT) with respect to core model components, multidisciplinary team (MDT) delivery, crisis response, safety management, and interagency working. The evaluation also aimed to identify shared principles and locally specific adaptations, including police integration within the Vancouver model.

**Methods:**

A descriptive, comparative service evaluation was conducted using a structured side-by-side framework across predefined domains: service context, implementation/operation, staffing/roles, and distinctive operational features. A scoping review and targeted grey-literature review identified established ACT components and local descriptions of intensive/assertive community care, including NHS England guidance and published descriptions of the Vancouver ACT model. Data were extracted into a comparison matrix (population focus, MDT composition, contact intensity, crisis/safety processes, and interagency interfaces) and synthesised narratively to describe similarities and differences.

**Results:**

Both services reflect core ACT principles reported in the literature, including MDT-based delivery and intensive, community-based support for people with severe mental illness and complex needs. NHS England guidance describes intensive and assertive community mental health care as a distinct, high-support form of community provision, consistent with the aims of assertive/outreach-style models. A key difference is Vancouver ACT’s formal integration of law enforcement, with designated Vancouver Police Department officers embedded within the team to enable coordinated responses. This embedded-police element represents a distinct approach to crisis intervention and safety management compared with standard UK community configurations described in national guidance.

**Conclusion:**

UK AOT-style services and Vancouver ACT share a common basis of MDT, high-intensity community care, but differ in how safety functions and interagency crisis working are structurally embedded. Further evaluation should compare outcomes (engagement, admissions, safety events, patient experience) and process measures (response times, multi-agency coordination) to determine benefits, risks, and acceptability across settings.

